# Resistance Exercise with Older Fallers: Its Impact on Intermuscular Adipose Tissue

**DOI:** 10.1155/2014/398960

**Published:** 2014-04-03

**Authors:** Janelle L. Jacobs, Robin L. Marcus, Glen Morrell, Paul LaStayo

**Affiliations:** ^1^Department of Physical Therapy, University of Utah, 520 Wakara Way, Salt Lake City, UT 84108, USA; ^2^Department of Radiology, University of Utah, 30 N 1900 E, Salt Lake City, UT 84132, USA

## Abstract

*Objective*. Greater skeletal muscle fat infiltration occurs with age and contributes to numerous negative health outcomes. The primary purpose was to determine whether intermuscular adipose tissue (IMAT) can be influenced by an exercise intervention and if a greater reduction in IMAT occurs with eccentric versus traditional resistance training. *Methods*. Seventy-seven older adults (age 75.5 ± 6.8) with multiple comorbidities and a history of falling completed a three-month exercise intervention paired with either eccentric or traditional resistance training. MRI of the mid-thigh was examined at three time points to determine changes in muscle composition after intervention. *Results*. No differences in IMAT were observed over time, and there were no differences in IMAT response between intervention groups. Participants in the traditional group lost a significant amount of lean tissue (*P* = 0.007) in the nine months after intervention, while participants in the eccentric group did not (*P* = 0.32). When IMAT levels were partitioned into high and low IMAT groups, there were differential IMAT responses to intervention with the high group lowering thigh IMAT. *Conclusions*. There is no decrease in thigh IMAT after a three-month exercise intervention in older adults at risk for falling and no benefit to eccentric training over traditional resistance training for reducing IMAT in these individuals.

## 1. Introduction

Aging is associated with increased fatty infiltration of skeletal muscle [1–5]. These age-associated changes in muscle composition, specifically elevated levels of intermuscular adipose tissue (IMAT), have been linked to a number of negative health consequences and functional impairments [5], including increased risk of hospitalization [6], mobility impairments [7, 8], strength deficits [1, 5], poor aerobic capacity [9], and poor performance on common clinical assessments, such as the 6-Minute Walk Test [10] and Five Times Sit-to-Stand Test [11]. Additionally, elevated levels of IMAT in older adults are positively associated with insulin resistance [12–14], perhaps due to the essential role of skeletal muscle in glucose disposal [13]. Because IMAT typically increases with age and may contribute to other morbidities that occur with advancing age, it is a priority to explore approaches by which IMAT levels may be reduced to diminish the personal and societal costs of age-associated skeletal muscle fat infiltration [5].

An inverse relationship exists between habitual physical activity of older adults and skeletal muscle fat infiltration [15–17], though the concept that older adults' level of IMAT is amenable to change with a focused rehabilitation program is less clear. The link between habitual physical activity levels and IMAT has been demonstrated in masters athletes [15], in diabetic older adults [16], and in older adults with peripheral arterial disease [17]. Only few studies, however, have demonstrated the effects of exercise interventions on IMAT. In one of these studies, Goodpaster et al. [18] demonstrated that a 12-month, multimodal exercise program prevented the age-associated increase in IMAT observed in an inactive control group during the intervention period. Decreases in IMAT have been reported in cohorts of healthy, community-dwelling older men and women after completing six months of multimodal exercise [19, 20]. Further, reductions in IMAT in community-dwelling older adults can occur with or without concomitant weight loss interventions coupled to multimodal physical activity programs [19]. Finally, strength training over 3–6 months has resulted in decreased levels of IMAT in elderly men and women [21, 22] and older adults with a variety of comorbid conditions [2]. However, the optimal type and intensity of resistance exercise as part of a multimodal rehabilitation program for reducing or preventing IMAT infiltration in an older, at-risk population is unknown. Additionally, since some have reported a differential muscle function response to exercise in those with high versus low initial levels of IMAT [23], it is curious if a differential gross muscle structure (change in IMAT) response occurs upon completion of a multimodal exercise program.

Several studies [18–20] have examined the effects of resistance training on IMAT as part of a multimodal exercise program in older adults, though only one has reported on the exclusive use of eccentric resistance training [2]. This heterogeneous cohort included survivors of stroke or cancer, individuals with impaired glucose tolerance or multiple sclerosis, and subjects who had undergone a total knee replacement, thereby making it difficult to generalize the IMAT results to a population of older adults participating in a focused rehabilitation intervention. Compared with traditional resistance exercise, which combines concentric and eccentric muscle contractions, resistance training exclusively using eccentric muscle contractions can result in higher levels of force production, making it a potentially higher intensity mode of resistance exercise [24]. This higher intensity eccentric training can also be performed at lower energetic costs and perceived exertion levels [24–26]. Several recent review papers [27–29] have characterized eccentric exercise as high intensity and documented its positive effect on muscle and rehabilitation outcomes. It is unknown, however, what effect eccentric training may have on IMAT as compared to traditional resistance exercise as part of a multimodal exercise program for older adults.

Therefore, the primary purpose of this study was to examine the effects on IMAT of eccentric resistance training, as compared to traditional resistance training, as part of a multicomponent exercise fall reduction program in older adults at risk for falls. The secondary purpose was to describe if the participants responded differently to resistance exercise in terms of changes in muscle composition when partitioned into high and low IMAT groups.

## 2. Methods

### 2.1. Participants and Time Points

**Figure 1:**
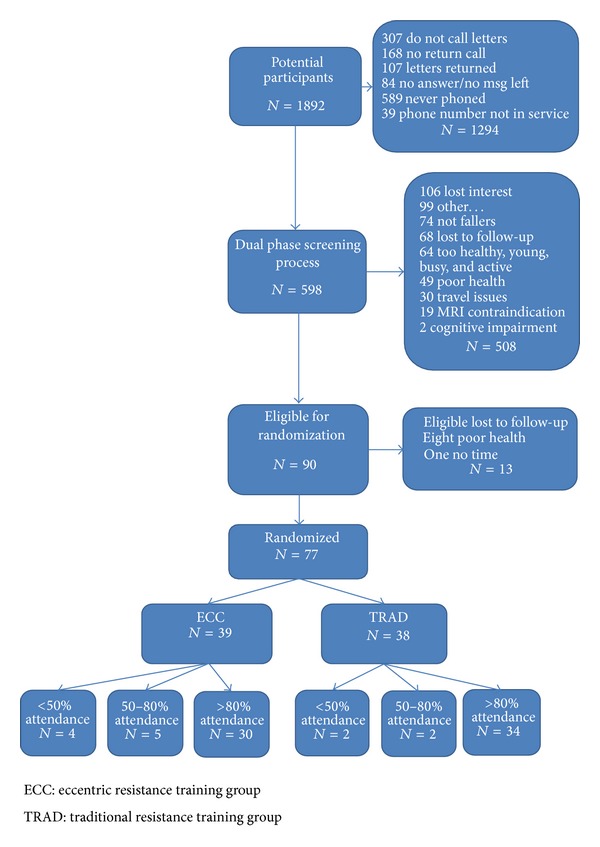
CONSORT flow diagram.

Seventy-seven older adults (75.5 ± 6.8 years, 20 males, and 57 females), who experienced an unintentional fall to the ground in the past year, volunteered to participate in this randomized multicomponent exercise fall reduction study that included either a traditional (TRAD) or eccentric (ECC) resistance training program. The participants were assessed on three occasions, at pretraining, posttraining, and at a nine-month follow-up time point. A block randomization process was used for assigning participants into either the ECC or TRAD groups (see CONSORT flow diagram, Figure 1). Participants were required to be ambulatory within the community with or without an assistive device yet they have two or more comorbid conditions. Written informed consent was obtained and the Institutional Review Board at the University of Utah approved the study procedures. Individuals with progressive neurological disorders, active cancer, chronic heart failure, or unstable medical conditions that precluded exercise were excluded. Baseline assessments included self-report of comorbidities and clinical measurements of gait speed and BMI.

### 2.2. Intervention

Participants trained for 60 minutes per session, three times per week for 12 weeks as part of a multicomponent exercise fall reduction program that included resistance training of the lower extremity. Training sessions consisted of multiple modes of exercises performed in a circuit that alternated higher intensity and dynamic activities with lower intensity, static tasks. Aerobic exercise was performed on a NuStep recumbent trainer (NuStep Inc, Ann Arbor, MI), seated stationary cycle ergometer, or overground treadmill. Flexibility exercises included pectoralis stretching in a doorway, seated hamstrings stretching, standing calf stretching, hooklying trunk rotations, and prone-on-elbows as tolerated. Balance exercises were performed with both static and dynamic bases of support and incorporated varied vestibular and visual inputs for altered sensory stimulation. Upper extremity resistance exercise was performed using free weights. The only difference in the multicomponent exercise fall reduction program was the type of lower extremity resistance training performed. There was no attempt at matching the workloads of the two resistance exercise (TRAD versus ECC) regimens, though the amount of time spent doing resistance exercise progressed in both groups to a maximum of 15 minutes. The TRAD group performed three sets of 15 repetitions of a seated bilateral leg press exercise (Tuff Stuff PS-230 Deluxe Leg Press, Tuffstuff, Chino, CA) at 60–65% of their one repetition maximum (RM) for the initial two weeks. Training sessions for the remaining 10 weeks were performed at 70% of 1-RM, which was assessed every two weeks thereafter. In addition, the TRAD group performed standing multidirectional straight leg exercises with a weighted cuff placed just proximal to the ankle. The training loads for this exercise were increased as tolerated every two weeks provided the participants could complete three sets of 15 repetitions. The ECC group performed a progressive resistive eccentric exercise of the knee and hip extensor muscles using a recumbent stepper-ergometer (Eccentron, Baltimore Therapeutic Equipment, Hanover, MD) as described previously [26, 29, 30]. Briefly, the stepper speed ranged between 12 and 18 revolutions per minute as the participant resisted the stepper pedal action and eccentric muscle contractions were induced in the knee and hip extensor muscles. Visual feedback of the work performed for each revolution was displayed on a computer monitor. Participants performed eccentric work from approximately 15–75 degrees of knee flexion as they resisted the motorized movement of the stepper pedals via resistance action of the knee and hip extensors. Perceived exertion was assessed with the Borg rating scale between 6 and 20 [31]. In the first week of ECC, sessions lasted three to five minutes and were performed at a “very, very light” intensity while resisting the stepper pedal action. During subsequent weekly training sessions, subjects were gradually allowed to resist the pedal action with more exertion as they progressed from a “fairly light” to a “somewhat hard” intensity level. The duration of each session was progressively increased to maximal 15 minute duration of ECC.

### 2.3. IMAT Determination

Magnetic resonance imaging (MRI) was used for determination of the cross-sectional area (CSA) of lean muscle mass and IMAT as has been done previously [7]. The primary outcome variable in this study was IMAT CSA. Bilateral MRI scans of the thighs were obtained and subjects were placed supine in a 3.0 Tesla whole body MR imager (Siemens Trio, Siemens Medical, Erlangen, Germany). The legs were scanned in a coronal plane and the midpoint of the thigh was determined and defined as half way between the superior margin of the femoral head and the inferior margin of the femoral condyles. Axial imaging (5 mm thick slices at 1 cm intervals) of the legs was then performed over half the length of the femur, centered at the midpoint of the thigh. Separate fat and water images were created with custom software using the three-point Dixon method [32]. A tissue model was then used to calculate estimates of total fat and nonfat volume fractions on a per-pixel basis, which were displayed in image form. A single image slice from the midpoint of each thigh was used to determine average cross-sectional area (cm^2^) of IMAT and lean tissue. Manual tracing eliminated subcutaneous fat and bone and isolated the fascial border of the thigh to create a subfascial region of interest (ROI). Total IMAT and lean tissues were calculated by summing the value of percent fat fraction and percent lean tissue fraction over all pixels within the ROI using custom-written image analysis software (MATLAB; The MathWorks, Natick, MA). This sum was multiplied by the area of each pixel to give total fat and lean tissue CSAs within the ROI and the respective IMAT and lean tissue cross-sectional areas were calculated after excluding subcutaneous adipose tissue and bone [32]. The same investigator blinded to group performed measurements of individual participants. This technique has demonstrated high levels of intrarater reliability [30], test-retest reliability [33], and concurrent validity when compared to imaging of a cadaveric phantom limb [30]. To normalize IMAT for thigh size, the percent of IMAT was calculated for each individual. This was done by dividing the area of IMAT (in cm^2^) by the overall area of the thigh (in cm^2^) excluding subcutaneous adipose tissue and bone. In the partitioning of the participants into a high or low IMAT category, participants from both intervention groups were stratified into high and low IMAT groups by percent fat (IMAT area/total muscle area) above or below the collective mean.

### 2.4. Statistical Analysis

To address the primary purpose of the study and to determine whether resistance training with ECC or TRAD induced thigh muscle composition (IMAT and lean changes), a two-way repeated measures analyses of variance (ANOVA) with the factors of group (ECC or TRAD) and time (pretraining, posttraining, and nine-month follow-up) was utilized at an alpha level of 0.05. The main effects and interaction effects were analyzed and pair-wise post hoc comparisons were employed when significant findings occurred. Separate one-way repeated measures analyses of variance were employed to determine within-group changes over time. Finally, to address the secondary purpose and determine whether there was a differential response to resistance exercise as an intervention by those with high or low IMAT content, a separate one-way repeated measures analysis of variance for the high and low IMAT groups, respectively, was used to assess changes in each group across each of three time points. The data was analyzed using SPSS version 20 (SPSS Inc, Chicago, IL).

## 3. Results

The ECC and TRAD intervention groups were not statistically different at baseline (see Table 1). There were no significant time (*P* = 0.89), group (*P* = 0.21), or interaction effects (*P* = 0.63) for changes in IMAT. A significant time effect for changes in lean (*P* = 0.007) occurred, though there were no significant group (*P* = 0.31) or group by time interaction (*P* = 0.21) effects for changes in lean. Within-group changes in each intervention group are shown in Table 2. There were no significant within-group changes in IMAT over time in either the ECC (*P* = 0.92) or TRAD (*P* = 0.64) groups. However, there was a significant (*P* = 0.007) within-group change in lean for the TRAD group, with the lean cross-sectional area at the nine-month follow-up being significantly lower than either the pretraining (*P* = 0.02) or posttraining (*P* = 0.05) values. There were no significant within-group changes in lean over time in the ECC group (*P* = 0.32). Neither the ECC (*P* = 0.51) nor the TRAD (*P* = 0.15) group demonstrated a significant change in BMI over time.

When stratifying the participants from both intervention groups by the relative amount of IMAT in their thighs (see Table 3), the high IMAT (14.5% or more) group demonstrated a loss of lean tissue CSA nine months after training (*P* = 0.04), while the low IMAT (14.4% or less) group did not change their lean tissue CSA (*P* = 0.18). Both the high and low IMAT groups, however, demonstrated changes in IMAT with the former losing (*P* = 0.014) and the latter gaining (*P* = 0.005) IMAT during the intervention. Further, the high IMAT group experienced a parallel lowering (*P* = 0.03) of their elevated pretraining BMI, while the low IMAT group's BMI did not change (*P* = 0.73). There was no difference in the number of self-reported comorbidities between the high and low IMAT groups at baseline (*P* = 0.39), although the high IMAT group did show a greater proportion of self-reported diabetes mellitus (*P* = 0.01) and hypertension (*P* = 0.008).

## 4. Discussion

The primary findings of this study indicate that a three-month, multimodal, fall prevention program for at-risk, community-dwelling, older adults did not induce a change in thigh IMAT when the program was paired with either eccentric or traditional resistance training. Further, thigh lean tissue area did not change during the intervention period, though a significant decline in lean mass was seen in the nine months following the conclusion of the exercise intervention. This decline in lean mass after the intervention period was greater in the traditional group than in the eccentric resistance training group.

Previous studies have examined the effects of multimodal exercise training on skeletal muscle composition in older adults and have shown that IMAT may have the capacity to change with specific exercise intervention protocols [19, 20, 22]. Other studies have shown favorable changes in IMAT with resistance training alone [2, 21]. However, some longitudinal studies have refuted this concept, citing no change in skeletal muscle fat infiltration with exercise [18, 34]. In one such study, Goodpaster et al. [18] suggested that a 12-month, multimodal, exercise intervention in a cohort of older adults prevented the increase in IMAT seen in their inactive control group over the same 12-month period. In the absence of a formal exercise intervention, a significant annualized increase in skeletal muscle fat infiltration was also reported in a sample of 1678 older adults from the Health ABC study cohort [3]. Therefore, the lack of change in IMAT over a one-year period in the present study may be a positive finding, although it is impossible to draw such a conclusion without a randomized, inactive control group for comparison.

A novel observation in the present study is that there may be a differential IMAT response to multimodal exercise interventions in older individuals at risk for falling. That is, participants characterized as high IMAT and a higher BMI responded differently than those whose initial levels of IMAT were low. This differential response to resistance exercise has been demonstrated in muscle function, that is, muscle quality [23], but not in muscle composition. When partitioned by the fraction of IMAT making up the mid-thigh cross-sectional area, participants with high IMAT content, defined as percent of mid-thigh IMAT above the mean, demonstrated a significant decline in both BMI and IMAT after multimodal exercise. It is possible that the decrease in IMAT observed in the high IMAT group is a reflection of that group's decrease in BMI. However, post hoc analysis of these changes shows that the significant change in IMAT occurred from pre- to posttraining (3-month duration), while the significant change in BMI for this group occurred from pretraining to the final follow-up (12-month duration). It is curious, therefore, that the changes in IMAT in the high IMAT group are not directly parallel to the changes in BMI. These observations suggest that the benefits of exercise for skeletal muscle composition may be greater for older adults with poorer muscle composition upon initiation of a multimodal exercise program. These benefits, however, apply only for the duration of a structured exercise intervention, since the older individuals considered to have high IMAT lost a significant amount of lean mass during the 9-month follow-up, while the low IMAT group did not.

This differential response of those with higher IMAT levels to the multimodal exercise intervention is a possible explanation for the lack of significant changes in IMAT seen in the two respective intervention groups. Other possible explanations include greater proportions of reported diabetes mellitus and hypertension in the high IMAT participants, the relatively short duration (three months) of the intervention compared to other studies (18-week to 12-month duration) [18–22] that favorably impacted IMAT levels, and the quantification of IMAT by MRI, which differentiates tissue types by signal characteristics [5, 35] rather than CT, which indirectly measures IMAT content by muscle attenuation [5, 36]. Additionally, our participants were at-risk fallers with an average of five comorbid conditions, which makes them fundamentally different from the healthy, moderately-functioning older adults included in similar studies [18, 21, 22]. These clinically distinct differences in the health of participants make these findings novel and may account for some of the discrepancies between our results and the results of previously published studies.

This study has several limitations. All subjects reported multiple comorbidities at baseline and a history of falls, so the results cannot be generalized to a population of nondisabled elderly. Further, the sample size was relatively small when separating the groups, and there was no inactive control group for comparison of results. Despite these limitations, there are clear conclusions drawn and some interesting questions raised.

An unexpected finding from this study was that participants with low initial IMAT demonstrated a significant increase in IMAT during the intervention period without concomitant change in BMI. Though one might expect individuals with low IMAT to have a more ideal muscle composition, the trend for both the high and low IMAT groups to converge toward an intermediate IMAT fraction with an exercise intervention introduces the question of whether there is an optimal level of IMAT that is desirable for aging skeletal muscle. Future studies should further examine this differential response of IMAT to exercise interventions, as well as other exercise interventions that may reduce the rate of fatty infiltration of skeletal muscle.

## 5. Conclusions

There is no decrease in thigh IMAT following a three-month, multicomponent exercise, fall reduction program in high fall-risk, older individuals and no differential impact of eccentric resistance training over traditional resistance training for muscle composition. The observed differential effects of training on those with higher amounts of IMAT at baseline provide an alluring direction for future study and should be confirmed.

## Figures and Tables

**Table 1:** Participant characteristics.

	Eccentric (*n* = 39)	Traditional (*n* = 38)	*P*
Gender (F : M)	29 : 10	27 : 11	0.74
Age (years)	76.2 (7.4)	74.6 (6.2)	0.32
BMI (kg/m^2^)	27.1 (4.8)	29.1 (6.2)	0.11
Gait speed (m/s)	1.14 (0.24)	1.10 (0.25)	0.42
Comorbidities	5.2 (2.2)	5.3 (2.0)	0.82

Mean (±SD); BMI: Body Mass Index; comorbidities: number of self-reported comorbid conditions at baseline.

**Table 2:** Muscle composition outcomes for eccentric and traditional resistance training groups.

	Eccentric group (*n* = 39)				Traditional group (*n* = 38)			
	Pretraining	Posttraining	Nine-month follow-up	Within-group change	Pretraining	Posttraining	Nine-month follow-up	Within-group change
IMAT (cm^2^)	29.53 (8.3)	29.56 (7.8)	29.33 (8.8)	*P* = 0.92	32.11 (11.4)	31.75 (10.2)	32.41 (9.3)	*P* = 0.64
Lean (cm^2^)	181.8 (34.2)	183.9 (34.1)	181.6 (31.2)	*P* = 0.32	193.1 (48.7)	193.9 (48.2)	188.6 (46.8)	*P* = 0.007

Eccentric group: participants who participated in eccentric resistance training as part of multimodal exercise program; traditional group: participants who participated in traditional resistance training as part of multimodal exercise program; lean: lean tissue cross-sectional area of mid-thigh; IMAT: intermuscular adipose tissue cross-sectional area of mid-thigh.

**Table 3:** Muscle composition outcomes for participants with high and low muscle fat fractions.

	High IMAT (*n* = 35)				Low IMAT (*n* = 42)			
	Pretraining	Posttraining	Nine-month follow-up	Within-group change	Pretraining	Posttraining	Nine-month follow-up	Within-group change
IMAT (cm^2^)	37.75 (8.8)	35.56 (8.8)	36.20 (8.6)	*P* = 0.014	25.01 (6.7)	26.53 (7.0)	26.38 (6.9)	*P* = 0.005
Lean (cm^2^)	181.6 (36.6)	183.2 (35.6)	178.6 (32.4)	*P* = 0.04	192.1 (46.1)	193.5 (46.1)	190.4 (44.4)	*P* = 0.18
% IMAT	17.24 (2.2)	16.27 (2.5)	16.88 (2.6)	*P* = 0.002	11.54 (1.6)	12.15 (2.4)	12.26 (2.2)	*P* = 0.012
BMI (kg/m^2^)	30.02 (5.3)	29.87 (5.1)	29.46 (5.2)	*P* = 0.03	26.42 (5.4)	26.42 (4.9)	26.24 (4.9)	*P* = 0.73

Mean (±SD); high IMAT >14.5% IMAT, low IMAT <14.5% IMAT; IMAT: intermuscular adipose tissue cross-sectional area; lean: lean tissue cross-sectional area; %IMAT: fraction IMAT per total lean and IMAT area of the mid-thigh; BMI: Body Mass Index.
